# Osilodrostat, a potent oral 11β-hydroxylase inhibitor: 22-week, prospective, Phase II study in Cushing’s disease

**DOI:** 10.1007/s11102-015-0692-z

**Published:** 2015-11-05

**Authors:** Maria Fleseriu, Rosario Pivonello, Jacques Young, Amir H. Hamrahian, Mark E. Molitch, Chikara Shimizu, Tomoaki Tanaka, Akira Shimatsu, Tracy White, Annie Hilliard, Chuan Tian, Nicholas Sauter, Beverly MK Biller, Xavier Bertagna

**Affiliations:** Northwest Pituitary Center, Departments of Medicine and Neurological Surgery, Oregon Health and Science University, Portland, OR USA; Dipartimento di Medicina Clinica e Chirurgia, Sezione di Endocrinologia, Università Federico II di Napoli, Naples, Italy; Department of Endocrinology, Hôpital Bicêtre, Université Paris-Sud, Assistance Publique Hôpitaux de Paris, Paris, France; Department of Endocrinology, Diabetes, and Metabolism, Cleveland Clinic Foundation, Cleveland, OH USA; Division of Endocrinology, Metabolism and Molecular Medicine, Northwestern University, Chicago, IL USA; Division of Laboratory and Transfusion Medicine, Hokkaido University Hospital, Sapporo, Japan; Division of Endocrinology and Metabolism, Chiba University Hospital, Chiba-city, Japan; Clinical Research Institute, National Hospital Organization Kyoto Medical Center, Kyoto, Japan; Novartis Pharmaceuticals Corporation, East Hanover, NJ USA; Neuroendocrine Clinical Center, Massachusetts General Hospital, Boston, MA USA; Department of Endocrinology, Centre de Référence des Maladies Rares de la Surrénale, Hôpital Cochin, Faculté de Médecine Paris Descartes, Université Paris 5, Paris, France

**Keywords:** LCI699, Osilodrostat, 11β-hydroxylase, Cushing’s, Cortisol

## Abstract

**Purpose:**

In a 10-week proof-of-concept study (LINC 1), the potent oral 11β-hydroxylase inhibitor osilodrostat (LCI699) normalized urinary free cortisol (UFC) in 11/12 patients with Cushing’s disease. The current 22-week study (LINC 2; NCT01331239) further evaluated osilodrostat in patients with Cushing’s disease.

**Methods:**

Phase II, open-label, prospective study of two patient cohorts. Follow-up cohort: 4/12 patients previously enrolled in LINC 1, offered re-enrollment if baseline mean UFC was above ULN. Expansion cohort: 15 newly enrolled patients with baseline UFC > 1.5 × ULN. In the follow-up cohort, patients initiated osilodrostat twice daily at the penultimate efficacious/tolerable dose in LINC 1; dose was adjusted as needed. In the expansion cohort, osilodrostat was initiated at 4 mg/day (10 mg/day if baseline UFC > 3 × ULN), with dose escalated every 2 weeks to 10, 20, 40, and 60 mg/day until UFC ≤ ULN. Main efficacy endpoint was the proportion of responders (UFC ≤ ULN or ≥50 % decrease from baseline) at weeks 10 and 22.

**Results:**

Overall response rate was 89.5 % (n/N = 17/19) at 10 weeks and 78.9 % (n/N = 15/19) at 22 weeks; at week 22, all responding patients had UFC ≤ ULN. The most common AEs observed during osilodrostat treatment were nausea, diarrhea, asthenia, and adrenal insufficiency (n = 6 for each). New or worsening hirsutism (n = 2) and/or acne (n = 3) were reported among four female patients, all of whom had increased testosterone levels.

**Conclusions:**

Osilodrostat treatment reduced UFC in all patients; 78.9 % (n/N = 15/19) had normal UFC at week 22. Treatment with osilodrostat was generally well tolerated.

**Electronic supplementary material:**

The online version of this article (doi:10.1007/s11102-015-0692-z) contains supplementary material, which is available to authorized users.

## Introduction

Cushing’s disease is caused by an adrenocorticotropic hormone (ACTH)-secreting pituitary tumor and is the most common cause of excess endogenous cortisol secretion [1–3]. Hypercortisolism can lead to substantial morbidity and premature death compared with the general population [4]. The primary treatment goals for Cushing’s disease are to normalize cortisol levels and reverse the signs and symptoms of hypercortisolism [2, 3]. First-line treatment is transsphenoidal surgery [2], although this is not always successful [5] and patients may relapse many years after apparent surgical success [6]. A number of medical therapies are currently used in clinical practice for the treatment of Cushing’s disease. These include pasireotide (multireceptor-targeted somatostatin analogue), cabergoline (dopamine receptor agonist), metyrapone and ketoconazole (adrenal steroidogenesis inhibitors), mitotane (adrenolytic agent) and mifepristone (glucocorticoid receptor antagonist) [3, 5, 7–18]. Since not all patients with Cushing’s disease achieve sufficient benefit with available therapies, there is a continuing need for new medical therapies.

Osilodrostat (LCI699) is an oral inhibitor of 11β-hydroxylase, which catalyzes the final step of cortisol synthesis. Although this mechanism of action is similar to that of metyrapone, osilodrostat has a longer plasma half-life (4–5 versus ~2 h), allowing twice-daily dosing (instead of 3–4 times daily), and is more potent against 11β-hydroxylase (in vitro IC_50_ of 2.5 versus ~7.5 nM for metyrapone), allowing for an overall more convenient dosing schedule. A proof-of-concept study (LINC 1; **L**CI **INC**ushing’s) demonstrated that osilodrostat normalized urinary free cortisol (UFC) levels in 11/12 patients with Cushing’s disease after 10 weeks [19]. The current Phase II study (LINC 2; clinicaltrials.gov NCT01331239) further assessed osilodrostat in patients with Cushing’s disease over a longer period of time and in more patients.

## Methods

### Participants

LINC 2 was a 22-week, prospective, open-label, multicenter, Phase II study (an expansion of the LINC 1 study by protocol amendment) that enrolled patients (aged 18–75 years) with a confirmed diagnosis of Cushing’s disease. Cushing’s disease was defined as: UFC levels above the upper limit of normal (ULN); a morning plasma adrenocorticotropic hormone (ACTH) level above the lower limit of normal (LLN); and evidence of a pituitary origin for the excess ACTH [based on: confirmation of a pituitary tumor of ≥6 mm by magnetic resonance imaging (MRI) with a positive dynamic test; history of inferior petrosal sinus (central to peripheral) gradient >3 after corticotropin-releasing-hormone or desmopressin stimulation; or histological confirmation of an ACTH-producing pituitary tumor in patients with previous pituitary surgery]. Patients were excluded from the study if they had: undergone major surgery within 1 month prior to screening; poorly controlled diabetes mellitus as evidenced by glycated hemoglobin (HbA_1c_) levels >9 %; compression of the optic chiasm; history of, or risk factors for, prolonged QTcF (Fridericia’s Correction Formula). The study was conducted in accordance with the Declaration of Helsinki, and an independent ethics committee or institutional review board for each study site approved the study protocol. All patients provided written informed consent to participate.

Patients were enrolled in two cohorts. The ‘follow-up cohort’ comprised patients who completed LINC 1. These patients were offered enrollment in LINC 2 if their current UFC level was above the upper limit of normal (ULN; i.e. >1 × ULN). Patients were off osilodrostat treatment for 15–19 months before enrollment in LINC 2 (administrative time between the end of LINC 1 and initiation of LINC 2). The ‘expansion cohort’ comprised newly enrolled patients who were naïve to osilodrostat and who were required to have UFC levels >1.5 × ULN. The UFC entry criterion was less strict for the follow-up than for the expansion cohort (osilodrostat naïve) because patients in the former had already met the criterion of UFC >1.5 × ULN in LINC 1.

### Study design and procedures

**Fig. 1:**
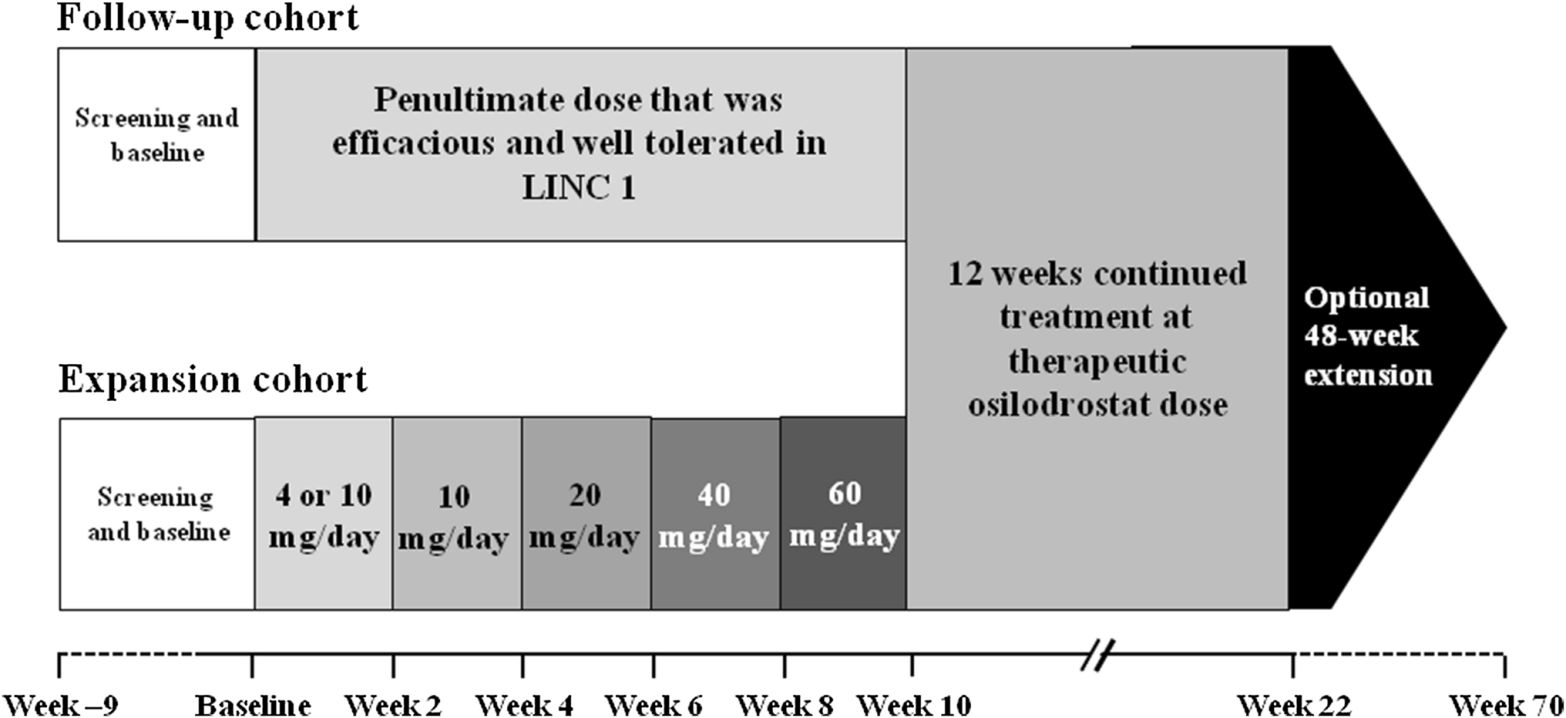
Study design and dosing schedule

The LINC 2 study design and dosing schedules are shown in Fig. 1; the screening period allowed adequate washout of any prior cortisol-lowering medications. Osilodrostat was given orally, twice daily (in the morning and in the evening, regardless of when the patient had eaten; no further specific instructions were given to the patients). In the follow-up cohort, osilodrostat was initiated at the penultimate dose that was efficacious and tolerable in LINC 1. If UFC remained >ULN, the dose was uptitrated from the penultimate dose according to the escalation sequence 10, 20, 40, and 60 mg/day until UFC was ≤ULN. This titration was continued up to week 10 as needed based on efficacy and tolerability. In the expansion cohort, osilodrostat was initiated at 4 mg/day if baseline UFC was >1.5 to ≤3 × ULN, and 10 mg/day if baseline UFC was >3 × ULN; dose was then escalated every 2 weeks according to the escalation sequence 10, 20, 40, and 60 mg/day until UFC was ≤ULN. If UFC normalized before week 10 in either cohort, dose was maintained at the effective level until week 10; if UFC normalized but subsequently increased to >ULN, dose escalation was resumed. If UFC was in the lower normal range or below the lower normal limit, and if the patient had symptoms suggestive of adrenal insufficiency, a dose reduction or interruption was considered. Patients completing the 22-week study could enter a 48-week extension phase if they responded to osilodrostat or were considered by the investigator to be receiving clinical benefit.

### Outcomes

The main efficacy endpoints were the proportion of patients who were controlled responders (mean UFC ≤ ULN), partially controlled responders (mean UFC > ULN and with ≥50 % reduction from baseline), or uncontrolled (mean UFC > ULN and with <50 % reduction from baseline) at weeks 10 and 22. Overall response rate was calculated as the sum of controlled and partially controlled patients. Baseline UFC measurements were based on the mean of three 24-h urine samples collected within 14 days before the first dose. During treatment, UFC measurements were based on the mean of at least two 24-h urine samples collected within 4 days before the relevant time point. UFC was measured at a central laboratory (Quest Diagnostics, Valencia, CA, USA) using liquid chromatography–tandem mass spectrometry (LC–MS/MS; normal range 11–138 nmol/24 h).

Secondary endpoints included changes from baseline to weeks 10 and 22 in various pharmacodynamic parameters, including serum (measured at 08:00) and salivary cortisol [measured in the morning (08:00) and late at night (23:00–24:00)], plasma ACTH, serum 11-deoxycortisol, plasma 11-deoxycorticosterone, plasma aldosterone, plasma renin, total serum testosterone, serum luteinizing hormone (LH), serum follicle-stimulating hormone (FSH), and serum estradiol. Escape from response, defined as mean UFC > ULN on at least two consecutive visits at the highest tolerated osilodrostat dose after previously attaining UFC normalization, was also evaluated. Safety and tolerability of osilodrostat treatment, as well as various clinical and laboratory parameters, were assessed throughout the study. Tumor size was assessed by MRI at baseline and 22 weeks. See Supplementary Appendix for further details.

### Statistical analyses

A sample size of 12–15 patients was required to provide 70–84 % power to reject the null hypothesis of a 15 % response rate when the alternative hypothesis of a 50 % response rate was true, based on an exact binomial test for a single proportion at a significance level of 0.05. This assumed that response rates of ≤15 % were unacceptable and that rates of ≥50 % were considered a good indication of a beneficial effect. The analysis was based on the mean UFC level at weeks 10 and 22, with response as a binary outcome. The proportions of patients who were responders at weeks 10 and 22 were summarized using point estimates and 95 % confidence intervals (CIs; exact method) and were evaluated separately for the follow-up and expansion cohorts, as well as for all patients combined. Patients who discontinued the study for a disease- or treatment-related reason, or whose mean UFC level at week 10 or 22 was >ULN and decreased by <50 % from baseline, were classified as non-responders or uncontrolled. The original planned analysis was defined by cohort. The analysis of all patients (pooled analysis of both cohorts combined) was not pre-specified in the protocol. Descriptive summaries and 95 % CIs were generated for the change from baseline to weeks 10 and 22 for cortisol levels and all pharmacodynamic, clinical, and laboratory parameters. Efficacy and safety data were calculated based on the safety analysis set, which comprised all patients who received at least one dose of study drug in LINC 2. Efficacy data are presented up to week 22 for each patient. Safety data are presented for a longer follow-up period, until the last patient had completed 22 weeks of treatment (data cut-off of 23 December 2013). See Supplementary Appendix for further details.

## Results

### Patient population

Between 7 January and 26 July 2013, 19 patients were enrolled: four in the follow-up cohort (starting dose: 4 mg/day, n = 1; 20 mg/day, n = 3) and 15 in the expansion cohort (starting dose: 4 mg/day, n = 9; 10 mg/day, n = 6; Table 1). Seventeen patients (89.5 %) completed the 22-week treatment period. One patient discontinued at the end of the 22-week treatment period, and 16 patients entered the optional extension phase.

**Table 1:** Patient demographics and baseline characteristics (safety analysis set)

	Follow-up cohort (n = 4)	Expansion cohort (n = 15)	All patients (n = 19)
Mean age ± SD, years	34.3 ± 5.5	37.5 ± 9.0	36.8 ± 8.4
Median (range)	35 (29–39)	36 (25–52)	36 (25–52)
Female:male, n	3:1	11:4	14:5
Race, n (%)			
Caucasian	4 (100.0)	11 (73.3)	15 (78.9)
Other	0	4 (26.7)	4 (21.1)
Median time since diagnosis (range), months	82.5 (57.6–100.3)	63.4 (12.2–155.2)	70.2 (12.2–155.2)
Previous surgery, n (%)	4 (100.0)	13 (86.7)	17 (89.5)
Mean baseline UFC ± SD, nmol/24 h^a^	398 ± 176^b^	1630 ± 3043	1371 ± 2734

*SD* standard deviation

^a^Normal range: 11–138 nmol/24 h

^b^3/4 patients had UFC > 1.5 × ULN at enrollment

### Response to osilodrostat

After 10 weeks of osilodrostat treatment, 84.2 % (n/N = 16/19) of patients were controlled and 5.3 % (n/N = 1/19) were partially controlled (Table 2); overall response rate was 89.5 % (n/N = 17/19). Two patients discontinued during the first 10 weeks, one because of a non-treatment-related administrative issue and one because of an adverse event (AE; grade 3 papular rash); see Supplementary Appendix for more details. At week 22, the overall response rate was 78.9 % (n/N = 15/19; Table 2); all responders were controlled responders. The details of the two patients who were responders at week 10 but not at week 22 are described in the Supplementary Appendix; one of these patients might have experienced an ‘escape’ from response.

**Table 2:** Proportion of UFC responders at weeks 10 and 22 (safety analysis set)

	Follow-up cohort (n = 4)	Expansion cohort (n = 15)	All patients (n = 19)
Week 10			
Responders, n (%) [95 % CI]	4 (100.0) [39.8, 100.0]	13 (86.7) [59.5, 98.3]	17 (89.5) [66.9, 98.7]
Controlled, n (%) [95 % CI]	4 (100.0) [39.8, 100.0]	12 (80.0) [51.9, 95.7]	16 (84.2) [60.4, 96.6]
Partially controlled, n (%) [95 % CI]	0 [0, 60.2]	1 (6.7) [0.2, 32.0]	1 (5.3) [0.1, 26.0]
Week 22			
Responders, n (%) [95 % CI]	3 (75.0) [19.4, 99.4]	12 (80.0) [51.9, 95.7]	15 (78.9) [54.4, 94.0]
Controlled, n (%) [95 % CI]	3 (75.0) [19.4, 99.4]	12 (80.0) [51.9, 95.7]	15 (78.9) [54.4, 94.0]
Partially controlled, n (%) [95 % CI]	0 [0, 60.2]	0 [0, 21.8]	0 [0, 17.7]

The most common total daily osilodrostat doses at week 22 were 10 mg/day (n = 4) and 20 mg/day (n = 5)

### Effect of osilodrostat on cortisol levels

**Fig. 2:**
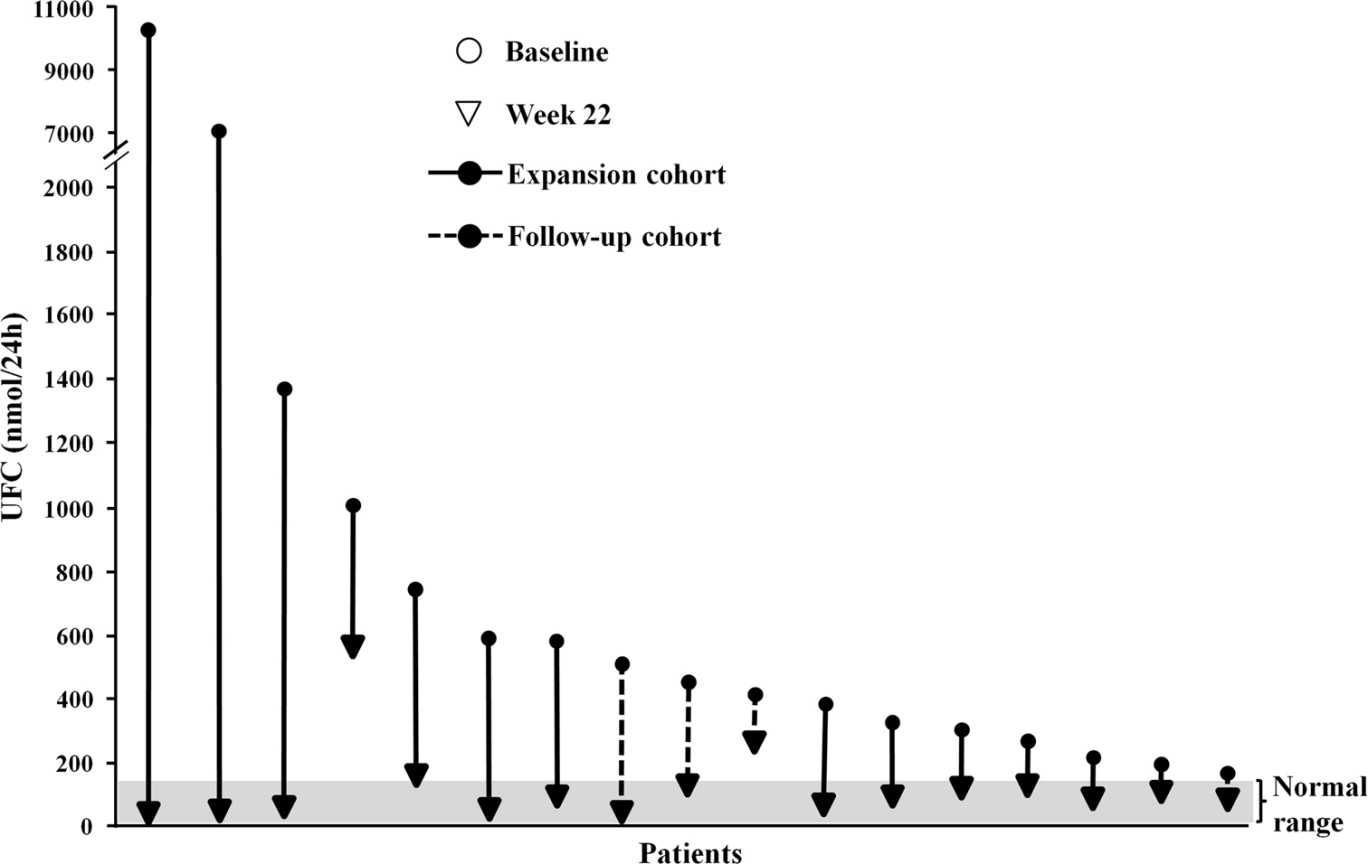
Absolute change in UFC from baseline in the 17 patients who completed 22 weeks (safety analysis set). Normal range: 11–138 nmol/24 h

**Fig. 3:**
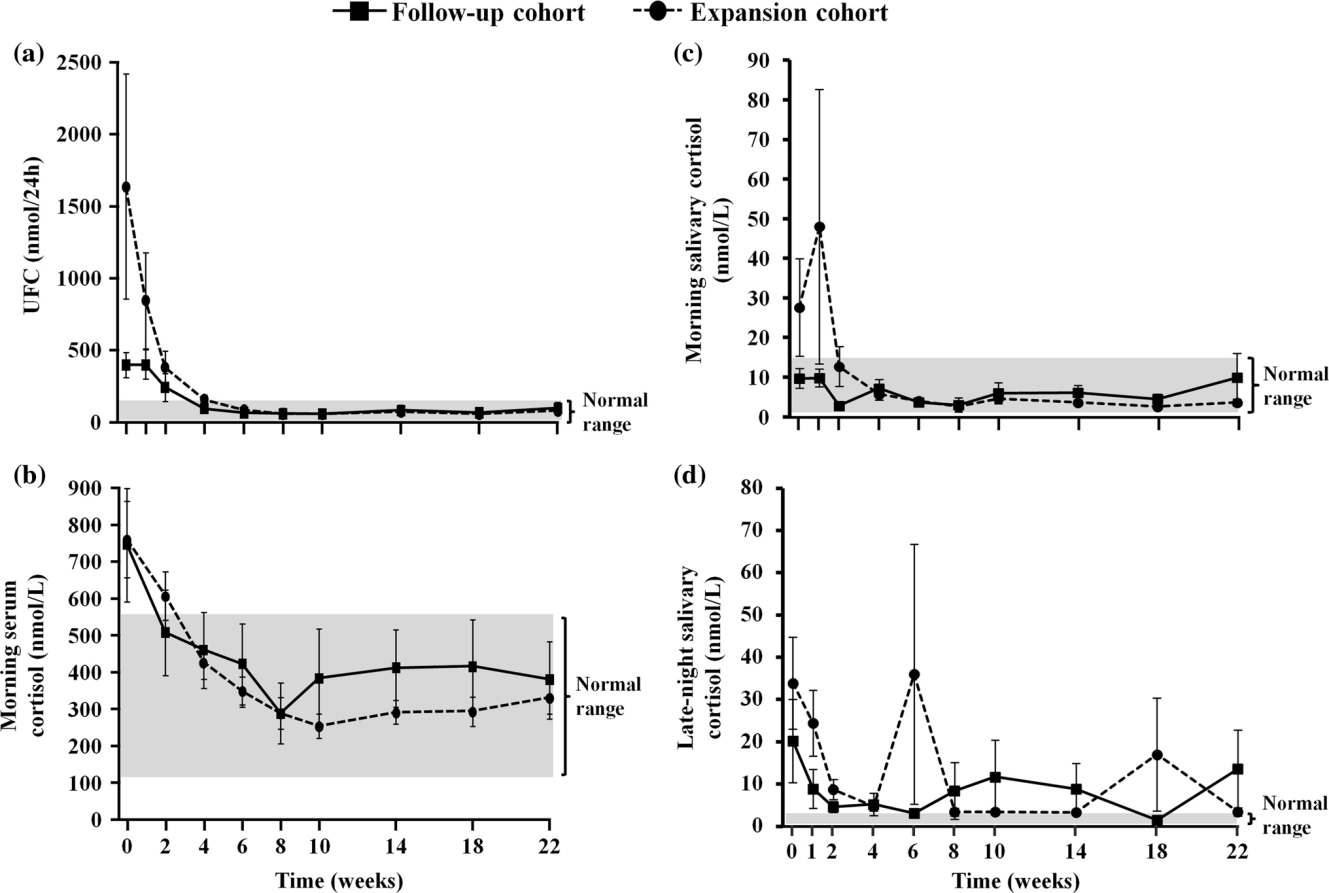
**a** UFC, **b** morning serum cortisol, **c** morning salivary cortisol, and **d** late-night salivary cortisol levels over time during osilodrostat treatment, by cohort (safety analysis set). All data are mean ± SE (standard error). Normal ranges are as follows: UFC, 11–138 nmol/24 h; morning serum cortisol, 127–567 nmol/L; morning salivary cortisol, 1.1–15.5 nmol/L; late-night salivary cortisol, ≤2.5 nmol/L

UFC levels decreased in all patients and were within the normal range in 15/17 patients (88.2 %) who reached week 22 (Fig. 2); decreases in the remaining two patients were 48.6 and 47.4 %. Overall mean UFC levels decreased rapidly from baseline (1371 ± 2734 nmol/24 h) to within the normal range by week 4 and remained suppressed through to week 22 (92 ± 124 nmol/24 h; Fig. 3); the decrease from baseline to week 22 was from 398 ± 176 to 98 ± 92 nmol/24 h in the follow-up cohort and from 1630 ± 3043 to 90 ± 136 nmol/24 h in the expansion cohort.

Changes in mean morning serum and salivary cortisol levels generally followed those of UFC, rapidly decreasing to within the normal range and remaining so until week 22 (Fig. 3). There was no change (from 10 ± 5 at baseline to 10 ± 11 nmol/L at week 22) in morning salivary cortisol levels in the follow-up cohort (levels were already within the normal range at baseline), while levels decreased from 28 ± 46 to 4 ± 3 nmol/L in the expansion cohort; the change for the overall population was from 24 ± 41 nmol/L at baseline to 5 ± 6 nmol/L at week 22. Late-night salivary cortisol levels also decreased, although the changes were more variable and measured levels remained above normal throughout treatment (Fig. 3). Of the 17 patients who completed the study: baseline serum cortisol levels were >ULN in 11 patients and, by week 22, levels had normalized in eight, remained >ULN in two, and fell to <LLN in one patient; baseline morning salivary cortisol levels were ≥ULN in five patients and, by week 22, had normalized in four patients and remained >ULN in one; baseline late-night salivary cortisol levels were >ULN in all 17 patients and, by week 22, had normalized in seven and remained >ULN in 10 patients.

### Effect of osilodrostat on other hormone levels

**Fig. 4:**
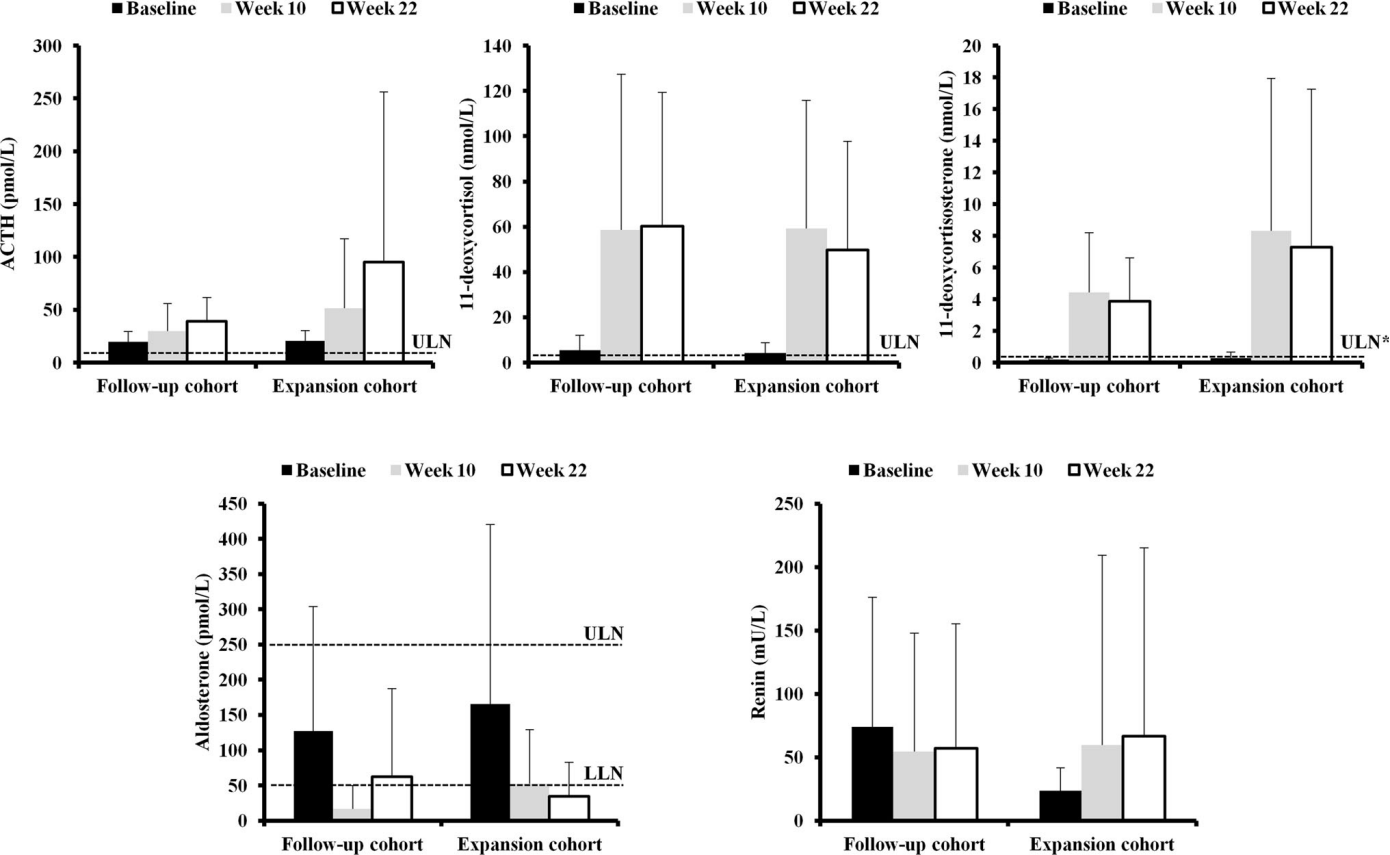
Hormone levels at baseline, week 10, and week 22, by cohort (safety analysis set). *Asterisk* indicated ULN is for females. All data are mean + SD. Normal ranges are as follows: ACTH, 1.8–9.2 pmol/L; 11-deoxycortisol, 0–3.92 nmol/L; 11-deoxycorticosterone, 0.12–0.35 nmol/L (males) and 0.05–0.39 nmol/L (females); renin, not available; aldosterone, 55–250 pmol/L

Mean baseline ACTH levels in the overall population were >ULN (20.2 pmol/L; normal range 1.8–9.2) and increased four-fold at week 22 (Fig. 4; Supplementary Table 1). The increase in ACTH was greater in the expansion than in the follow-up cohort (Supplementary Figure 1) and was primarily driven by two patients; see Supplementary Appendix for further details.

Overall mean baseline 11-deoxycortisol levels were 4.5 nmol/L (normal range 0–3.92) and levels increased markedly (11-fold at week 22) during treatment (Fig. 4; Supplementary Table 1). Similarly, overall 11-deoxycorticosterone levels increased (24-fold) and were >ULN at week 22 (6.3 nmol/L; normal range 0.05–0.39) (Fig. 4; Supplementary Table 1).

Overall mean aldosterone levels were within the normal range at baseline (157 pmol/L; normal range 55–250) and decreased during treatment (Fig. 4; Supplementary Table 1); mean levels were below the LLN at week 22 (41.7 pmol/L). Notably, renin levels decreased in the follow-up cohort (0.8-fold to 57.1 ± 98.2 mU/L) and increased in the expansion cohort (2.8-fold to 66.6 ± 148.7 mU/L) during osilodrostat treatment (Fig. 4; Supplementary Table 1).

At baseline, mean testosterone levels in female patients were 1.2 ± 0.7 nmol/L; levels increased to >ULN during treatment (4.0 ± 3.4 nmol/L at week 22; normal range 0.1–1.6 nmol/L). Baseline levels were >ULN in 5/14 (35.7 %) female patients, all of whom had post-baseline values >ULN. Of the 12 female patients who completed 22 weeks, testosterone levels at week 22 were >ULN in nine (75.0 %) (Supplementary Figure 2). New or worsening hirsutism (n = 2) and/or acne (n = 3) were reported among four female patients during the study, all of whom had increased testosterone levels. Baseline mean testosterone levels in males were slightly below normal (7.4 ± 3.5 nmol/L; normal range 8.7–38.2) and increased to within the normal range during osilodrostat treatment (13.2 ± 5.7 nmol/L at week 22). At baseline, two males had low testosterone levels and three had levels slightly greater than LLN (Supplementary Figure 2); during treatment, all male patients had increases to the mid-normal range. See Supplementary Appendix for data on estradiol, LH, and FSH (Supplementary Table 2).

### Changes in clinical and laboratory parameters

Mean body weight (–1.5 ± 3.8 kg) and body mass index (–0.5 ± 1.4 kg/m^2^) in the overall population did not show a clinically meaningful change from baseline to week 22 (Table 3). Of the 17 patients who completed the study, 12 had a decrease in weight and five had an increase. Edema (generalized and peripheral) was reported as an AE in two patients. One patient had a weight gain from 127 kg on day 1 to 137 kg on day 70 and lower extremity swelling on day 86. The other patient had a history of diabetes insipidus and reported generalized edema on day 31; weight was 129 kg on day 1 and 127 kg on day 28. Both patients had similar 11-deoxycorticosterone levels to the population mean and no history of congestive heart failure. However, the first patient had a history of intermittent lower extremity swelling since 2001.

**Table 3:** Changes in clinical/laboratory parameters during osilodrostat treatment in the overall population (safety analysis set)

Parameter	Baseline (n = 19)	Week 22 (n = 17)	Absolute change from baseline	Percentage change from baseline
Weight, kg	85.1 ± 24.0	85.6 ± 26.2	–1.5 ± 3.8	–3.0 (–7, 6)
Body mass index, kg/m^2^	30.7 ± 7.0	30.1 ± 7.9	–0.5 ± 1.4	–3.1 (–7, 7)
Systolic blood pressure,^a^ mmHg	132.6 ± 11.6	131.9 ± 17.8	–1.0 ± 16.2	–0.5 (–20, 26)
Patients with baseline^b^ hypertension (n = 13)	133.6 ± 13.1	133.5 ± 20.1	–0.8 ± 19.0	–4.9 (–20, 26)
Diastolic blood pressure,^a^ mmHg	85.1 ± 6.5	86.0 ± 8.9	1.3 ± 9.7	2.4 (–15, 24)
Patients with baseline^b^ hypertension (n = 13)	85.4 ± 7.5	86.8 ± 9.7	1.9 ± 11.2	2.4 (–15, 24)
Fasting plasma glucose, mg/dL	105.6 ± 49.0	81.2 ± 9.0	–14.9 ± 28.9	–10.2 (–58, 18)
Patients with baseline^b^ diabetes mellitus (n = 8)	133.4 ± 67.2	82.7 ± 12.3	–33.3 ± 41.0	–21.4 (–58, –5)
HbA_1c_, %	5.7 ± 0.7	5.5 ± 0.6	–0.2 ± 0.3	–2.2 (–11, 8)
Patients with baseline^b^ diabetes mellitus (n = 8)	6.4 ± 0.5	6.0 ± 0.5	–0.3 ± 0.3	–5.5 (–11, 0)
Total cholesterol, mmol/L	5.3 ± 1.4	4.6 ± 0.8	–0.7 ± 1.4	–8.0 (–39, 70)
Patients with baseline^b^ dyslipidemia (n = 6)	5.9 ± 1.8	5.2 ± 0.9	–0.7 ± 1.9	–12.6 (–39, 70)
HDL-cholesterol, mmol/L	1.7 ± 0.9	1.3 ± 0.4	–0.5 ± 0.8	–16.6 (–68, 11)
Patients with baseline^b^ dyslipidemia (n = 6)	1.5 ± 0.5	1.3 ± 0.6	–0.2 ± 0.2	–13.7 (–34, 11)
LDL-cholesterol, mmol/L	3.3 ± 1.7	2.8 ± 0.6	–0.6 ± 1.6	–15.2 (–57, 350)
Patients with baseline^b^ dyslipidemia (n = 6)	3.6 ± 1.9	3.1 ± 0.7	–0.5 ± 1.7	–17.8 (–48, 350)
Triglycerides, mmol/L	1.5 ± 0.6	1.3 ± 0.6	0 ± 0.4	–11.9 (–38, 65)
Patients with baseline^b^ dyslipidemia (n = 6)	1.7 ± 0.7	1.7 ± 0.4	–0.1 ± 0.6	–7.8 (–29, 44)

All data are mean ± SD, except for percentage change data, which are median (minimum, maximum). Normal ranges are as follows: fasting plasma glucose, 70–110 mg/dL; HbA_1c_, < 6.4 %; total cholesterol, 3.9–6.5 mmol/L; HDL-cholesterol, 1–1.7 mmol/L; LDL-cholesterol, 0–4.2 mmol/L; triglycerides, 0.6–1.7 mmol/L

*HDL* high-density lipoprotein, *LDL* low-density lipoprotein

^a^The highest reported systolic blood pressure measurement was 174 mmHg; the highest reported diastolic blood pressure measurement was 103 mmHg

^b^Refers to ‘a history of’ at baseline

There was little mean change from baseline in systolic or diastolic blood pressure at week 22, either in the overall population (–1.0 ± 16.2 and 1.3 ± 9.7 mmHg, respectively) or in the 13 patients with a baseline history of hypertension (–0.8 ± 19.0 and 1.9 ± 11.2 mmHg, respectively; Table 3). Five patients who completed the study had elevated baseline systolic blood pressure (defined as >139 mmHg [20]); levels normalized at week 22 in two patients and remained elevated in three. One patient with normal baseline levels had elevated systolic blood pressure at week 22. Four patients who completed the study had elevated diastolic blood pressure at baseline (defined as >89 mmHg); levels remained elevated at week 22 in all four patients. Another four patients with normal baseline levels had elevated diastolic blood pressure at week 22. There were no notable increases in systolic (defined as ≥180 mmHg) or diastolic (defined as ≥105 mmHg) blood pressure. One patient was reported to have an AE of hypertension.

Decreases from baseline to week 22 were observed in fasting plasma glucose (FPG; –14.9 ± 28.9 mg/dL) and HbA_1c_ levels (–0.2 ± 0.3 %) (Table 3); the improvements in FPG were greater (–33.3 ± 41.0 mg/dL) in the eight patients who had a baseline history of diabetes mellitus. There were also clinically relevant decreases to within the normal range in cholesterol and triglyceride levels from baseline to week 22 (Table 3). There were no clinically relevant changes in mean vital signs [although one patient had a notably elevated pulse rate (defined as ≥120 bpm) on one occasion during treatment] or notable electrocardiogram measurements over the study period (except for the patient with a reported serious AE of QT prolongation). Overall, there were no clinically meaningful changes from baseline to week 22 in mean sodium (140.6 ± 2.7 to 141.0 ± 2.1 mmol/L) or potassium (4.1 ± 0.4 to 3.8 ± 0.4 mmol/L) levels. Based on laboratory assessment, nine patients developed mild hypokalemia (range 3.0–3.4 mmol/L), although only one case was reported as an AE by the investigator; two patients with hypokalemia received potassium supplementation. One patient developed hyperkalemia; a laboratory value of 4.6 mmol/L (laboratory normal range was 3.5–4.5 mmol/L) was reported 2 weeks after initiation of osilodrostat therapy and it was reported as an AE 15 days later. Potassium levels were subsequently within the normal range from week 4 to week 22.

### Changes in pituitary tumor size

Data on each individual patient with measurable tumor size are summarized in Supplementary Table 4. Tumor size was not evaluable in 13/19 patients because the tumor was too small to be visualized, or anatomical changes post-pituitary surgery and/or radiation obscured the measurement. Two patients discontinued from the study before week 22 and therefore had no follow-up imaging. In the six patients with measurable tumors (Supplementary Table 3), diameter changes of 1.0–1.7 mm were observed from baseline to week 22; these changes are not considered to be clinically meaningful (i.e. <2.0 mm [21–23]). Two patients had an increase in the maximal tumor diameter at week 22 (range 1.0–1.7 mm). One patient had no change, and three patients had a decrease in maximal tumor diameter (1.0 mm each). See Supplementary Appendix for further details. Only one patient had measureable tumor volume at both baseline and week 22; volume increased from 13.7 mm^3^ at baseline to 17.5 mm^3^ at week 22 (change of 3.8 mm^3^, +28 %), which suggests a clinically meaningful change [23–25].

### Safety and tolerability of osilodrostat

Safety was assessed over a median period of 26.7 weeks’ treatment (range 2–50). Nearly all patients (18/19; 94.7 %) experienced at least one AE; the AEs most commonly reported by the investigator are shown in Table 4.

**Table 4:** Most common AEs (≥5 patients overall) reported during osilodrostat treatment, regardless of study drug relationship (safety analysis set)

All patients (n = 19)	All grades, n (%)	Grade 3–4,^a^ n (%)
Clinical AEs		
Nausea	6 (31.6)	0
Diarrhea	6 (31.6)	0
Asthenia	6 (31.6)	0
Adrenal insufficiency	6 (31.6)	1 (5.3)
Nasopharyngitis	5 (26.3)	0
Laboratory AEs		
Testosterone increased	5 (26.3)	1 (5.3)
Adrenal precursors increased	7 (36.8)	0
ACTH increased	6 (31.6)	0

^a^Severity grades assessed by National Cancer Institute Common Terminology Criteria, version 4.03

Three serious AEs were reported in two patients: one patient had QT prolongation (suspected to be drug related) in the context of an acute hospitalization for a serious AE of gastroenteritis (not suspected to be drug related) with dehydration; the other patient had uncontrolled Cushing’s disease as reported by the investigator (not suspected to be drug related); see Supplementary Appendix for further details. Adrenal insufficiency was reported as an AE in six patients. Mean UFC and morning serum cortisol values were <LLN at the time the AE was reported in four and three of the six patients, respectively (Supplementary Table 4); no other patients had UFC < LLN during the study. Osilodrostat treatment was decreased in five patients with adrenal insufficiency (two of these patients also had treatment interrupted at a different time point), and one further patient received replacement therapy with dexamethasone. One patient had syncope associated with adrenal insufficiency; no arrhythmia was documented in this patient.

## Discussion

The LINC 2 study demonstrated that osilodrostat, a potent oral 11β-hydroxylase inhibitor, decreased UFC levels in all patients with Cushing’s disease and maintained normal UFC in 79 % of patients (n/N = 15/19) after 22 weeks of treatment. Of the remaining four patients, two had ~50 % decreases in UFC—though it is important to note that normalization of cortisol levels is the goal of treatment—and two discontinued the study (one because of an AE, the other as a result of an administrative issue). Two patients were responders at week 10, but not at week 22; there is a possibility that one of these patients ‘escaped’ from response (see Supplementary Appendix). Notably, all enrolled patients had normalized UFC levels at least once during the study. Overall, these data confirm, over a longer follow-up period, the results from the LINC 1 study in which 11/12 patients (92 %) had normalized UFC after 10 weeks [19]. Taken together, these data demonstrate that osilodrostat effectively controls UFC in patients with Cushing’s disease. Changes in late-night salivary cortisol levels were variable and remained above normal throughout treatment; at this time, we do not know whether the exact timing of the evening osilodrostat dose might have impacted on the changes in late-night salivary cortisol.

Osilodrostat treatment led to expected increases above normal in 11-deoxycortisol and 11-deoxycorticosterone; these increases are in line with observations in LINC 1 [19]. It is worth noting that in LINC 1, levels of 11-deoxycortisol and 11-deoxycorticosterone decreased towards baseline following 14 days of osilodrostat washout; there was no such washout period in LINC 2. ACTH levels also increased in LINC 2, possibly as a compensatory reaction to the reduction in serum cortisol levels. The large increase in ACTH levels in the expansion cohort was primarily driven by two patients who had dramatic increases. Renin levels changed in opposite directions in the two cohorts. This is difficult to explain, but it may be a chance finding related to the small patient numbers, particularly in the follow-up cohort, and the large variability in the observed data. In addition, an effect of concomitant medications (e.g. diuretics such as spironolactone or eplerenone) on renin levels cannot be excluded.

Mean body weight was relatively unchanged throughout osilodrostat treatment in LINC 2; a possible effect of the mineralocorticoid precursors on body weight through fluid retention cannot be excluded. By contrast, in LINC 1, there was an increase in mean weight of 3.5 kg in the overall population [19]. Although the change in LINC 1 was primarily due to a single patient who experienced a 19 kg increase, there remained an overall mean increase of 2.4 kg when this patient was not included. There was no change in blood pressure in the current study, whereas there was a trend towards an improvement in LINC 1. However, baseline blood pressure levels in LINC 2 were generally lower than in LINC 1, which may explain the difference between the studies. Another possibility is that the increase in mineralocorticoid precursors may have offset the blood-pressure-lowering effect of inhibiting aldosterone synthesis (see Fig. 4). In the overall study population, there were modest improvements in glucose and HbA_1c_ levels during osilodrostat treatment; the improvements were more substantial in patients with a history of diabetes mellitus at baseline. There were also modest decreases in cholesterol (total, HDL, and LDL) and triglyceride levels; the reduction in HDL-cholesterol is an unfavorable effect and, in women, may be related to increased testosterone levels [26]. The possibility that the effects of concomitant medications (e.g. for hypertension, diabetes, or dyslipidemia) may have impacted changes in these clinical parameters cannot be excluded. An analysis of the relationship between such medications and associated clinical outcomes is planned in future studies. There were no significant changes in mean potassium levels, although nine patients developed mild hypokalemia and one patient developed borderline hyperkalemia (2 weeks after initiating osilodrostat therapy) based on laboratory assessments. Only one case of hypokalemia was reported as an AE. The mild abnormalities in serum potassium levels were transient and managed effectively with potassium supplements or resolved without intervention. In the overall population, mean baseline levels of fasting plasma glucose, HbA_1c_, cholesterol and triglycerides were within the normal range. Therefore, it is perhaps not surprising that no substantial changes were observed during osilodrostat treatment, despite the improvements in UFC levels; longer-term studies evaluating the effect of osilodrostat on changes in clinical and laboratory parameters in a larger number of patients are required. In addition, patients who had diabetes mellitus and dyslipidemia at baseline appeared to be well controlled, since they also had fasting plasma glucose/HbA_1c_ and cholesterol/triglyceride levels, respectively, within the normal range at week 22.

Osilodrostat treatment was generally well tolerated; AEs were consistent with those observed in LINC 1. Only one patient discontinued because of an AE, and 16/17 patients who completed LINC 2 elected to continue in the optional extension, which is ongoing; this suggests a high level of patient acceptance of the medication in this small patient population. Adrenal insufficiency was reported as an AE in six patients; the symptoms may alternatively have been a result of glucocorticoid withdrawal syndrome in some patients. The observation of adrenal insufficiency and/or steroid withdrawal in approximately one-third of patients highlights the potency of osilodrostat. It may therefore be prudent in future studies to titrate the dose of osilodrostat more slowly than was done in this dose-escalation study, and to target UFC levels in the mid-normal range.

Serial pituitary imaging was performed to screen for the theoretical risk of corticotroph tumor progression, analogous to Nelson’s syndrome in patients with bilateral adrenalectomy [21]. Although there was minimal change (<2.0 mm in maximal diameter) in tumor diameter during the period of observation in this study in the six patients with measurable tumors at baseline and week 22, it is important to note that longer follow-up is needed to explore more fully changes in tumor volume during osilodrostat treatment.

One potential effect of increased testosterone levels is hirsutism in women. No hirsutism was reported in LINC 1 despite significantly increased testosterone levels, although the authors speculated that this was because the short study duration (10 weeks) may have been insufficient to observe such effects [19]. In this 22-week study, testosterone levels increased in the female population, and new or worsening hirsutism (n = 2) and/or acne (n = 3) were reported among four female patients, all of whom had testosterone levels >ULN at week 22. Most increases in testosterone in female patients were only moderate, except for one patient who had a >10-fold increase (to ~16 nmol/L). Interestingly, this patient was not one of the four to report acne or hirsutism as an AE. Testosterone levels in male patients increased from sub- or low-normal levels to the mid-normal range, suggesting that osilodrostat may have a potential therapeutic effect. In general, data on gonadotroph function and possible clinical effects of androgen increases are limited in this Phase II study, but may be evaluated in greater detail in future studies of osilodrostat.

It should be noted that these data are somewhat limited by the fact that LINC 2 is an expanded, open-label, uncontrolled study, conducted in a small number of patients, some of whom (4/19) had previously received osilodrostat. A confirmatory Phase III study (LINC 3) is ongoing to evaluate the effect of osilodrostat in a larger patient population. This and other future studies will determine the place of osilodrostat in the medical treatment of Cushing’s disease.

In conclusion, osilodrostat demonstrated good efficacy with a satisfactory safety profile in this Phase II study, showing promise for the future treatment of patients with Cushing’s disease.

## Electronic supplementary material

Supplementary material 1 (DOCX 224 kb)
