# Supplementary material for: Osilodrostat, a potent oral 11β-hydroxylase inhibitor: 22-week, prospective, Phase II study in Cushing’s disease
Source: Pituitary. 2015 Nov 5;19:138–48. doi: 10.1007/s11102-015-0692-z (PMC4799251; doi:10.1007/s11102-015-0692-z)
Supplement: Supplementary file 1 — Supplementary material 1 (DOCX 224 kb) [file 11102_2015_692_MOESM1_ESM.docx]

**Osilodrostat, a potent oral 11β-hydroxylase inhibitor:**

**22-week, prospective, Phase II study in Cushing’s disease**

Maria Fleseriu MD, Rosario Pivonello MD, Jacques Young MD,
Amir H Hamrahian MD, Mark E Molitch MD, Chikara Shimizu MD,
Tomoaki Tanaka MD, Akira Shimatsu MD, Tracy White MSc, Annie Hilliard PhD,
Chuan Tian PhD, Nicholas Sauter MD, Beverly MK Biller MD, Xavier Bertagna MD

**Supplementary Appendix**

**Methods**

**Outcomes**

Pharmacodynamic parameters were analyzed at one of two central laboratories (Quest Diagnostics, Hounslow, UK or Quotient, Fordham, UK) and were assessed using liquid chromatography–tandem mass spectrometry (LC-MS/MS; serum cortisol, salivary cortisol, 11-deoxycortisol, 11-deoxycorticosterone, aldosterone), radioimmunoassay (renin), or chemiluminescent immunoassay (ACTH). Of the parameters measured, Quest Diagnostics analyzed: UFC, cortisol, ACTH, 11-deoxycortisol, aldosterone, renin, total testosterone, luteinizing hormone (LH), follicle-stimulating hormone (FSH), estradiol, and HbA_1c_. Quotient analyzed: UFC, salivary cortisol, 11­deoxycorticosterone, potassium, and sodium. The primary endpoint is based on the UFC measurements assessed at Quest Diagnostics.

Safety and tolerability were assessed based on the monitoring and recording of all adverse events (AEs) throughout the study. AEs were defined using terminology in the Medical Dictionary for Regulatory Activities (MedDRA; version 13.1), and severity was graded according to the National Cancer Institute Common Toxicity Criteria version 4.03. Various clinical and laboratory parameters, such as blood pressure, weight, fasting plasma glucose, HbA_1c_, and fasting lipid levels, were assessed after 10 and 22 weeks of treatment. Other safety assessments included: physical examination (for clinical signs of hyper- or hypocortisolism and possible AEs related to osilodrostat); safety electrocardiograms 1.5 hours post-dose (for QTcF prolongation) at each visit; MRI scan of pituitary with contrast (for evidence of possible corticotroph tumor progression) at screening/baseline and week 22; electrolytes, blood urea nitrogen, creatinine, liver function tests, hematology, biochemistry, and urinalysis at each visit (for general safety, hypokalemia, or hyperkalemia); serum or urine pregnancy test (women only) at each visit.

**Results**

**Patient population**

Two patients discontinued prematurely from the study. One patient (expansion cohort) discontinued at week 2 because of an AE (grade 3 papular rash); muscular weakness, nausea, and diarrhea were also reported. UFC levels had decreased rapidly from 205 to 54 nmol/24h during the week prior to discontinuation. The other patient (expansion cohort) discontinued at week 6 because of a non-treatment-related administrative issue. This patient did not report any symptomatic AEs and had UFC levels within the normal range at discontinuation. Although both patients who discontinued had UFC <ULN, they are classified as non-responders because they were not in the study at weeks 10 or 22.

**Response to osilodrostat**

*Partial responders and non-responders*

Two patients classified as non-responders at week 22 were responders at week 10. The first of these was the patient hospitalized for a serious AE of polyuria/polydipsia with uncontrolled Cushing’s disease during week 14. This patient had been classified as a partial responder at week 10 since UFC levels had decreased by >50% from baseline. Osilodrostat dose was titrated to 20 mg/day at week 2 and the patient had a normal UFC at week 8 on that dose. Dose was subsequently reduced at week 8 to 10 mg in the morning and 5 mg in the evening (owing to loss of appetite, asthenia, headache, and nausea); there were no further dose changes to week 22, and all UFC values between week 8 and week 22 were >ULN. Thus, although a dose of 20 mg/day resulted in control, a dose reduction to
15 mg/day was performed because of AEs and UFC levels were no longer controlled on the lower dose. The second patient had UFC levels <ULN at weeks 6, 10, and 18, but not at week 14 or 22, based on analyses at the central laboratory. However, based on analyses at the local laboratory, UFC levels were <ULN at weeks 6, 10, 14, 18, and 22. As such, there was a discrepancy between the laboratory values at week 14. As the local laboratory value, which was being used for the purpose of dose adjustments, was normal at week 14, the dose was not increased. Osilodrostat dose was titrated to 10 mg/day at week 2 and this dose was unchanged until week 22.

**Effect of osilodrostat on other hormone levels**

**Supplementary Table 1. ACTH and adrenal hormones during osilodrostat treatment (safety analysis set)**

|  | **Follow-up cohort** | | | **Expansion cohort** | | | **All patients** | | |
| --- | --- | --- | --- | --- | --- | --- | --- | --- | --- |
|  | **Baseline** | **Week 10** | **Week 22** | **Baseline** | **Week 10** | **Week 22** | **Baseline** | **Week 10** | **Week 22** |
| ACTH, pmol/L | 19.8 ± 9.8  (12–34) | 29.8 ± 26.0  (5–59) | 39.3 ± 22.5  (16–60) | 20.4 ± 9.8  (6–43) | 51.5 ± 65.5  (11–246) | 95.0 ± 161.1  (16–581) | 20.2 ± 9.5 (6–43) | 46.1 ± 58.1 (5–246) | 81.1 ± 140.6 (16–581) |
| 11-deoxycortisol, nmol/L | 5.5 ± 6.5 (1.3–15.2) | 58.5 ± 68.9 (5.1–158.0) | 60.2 ± 59.2 (8.4–145.5) | 4.2 ± 4.6  (0–19.1) | 59.3 ± 56.6 (4.8–179.1) | 49.9 ± 47.9 (7.0–168.3) | 4.5 ± 4.9  (0–19.1) | 59.1 ± 57.4  (4.8–179.1) | 52.4 ± 49.1  (7.0–168.3) |
| 11-deoxycortico-sterone, nmol/L | 0.2 ± 0.1  (0.1–0.3) | 4.4 ± 3.8  (0.3–9.0) | 3.9 ± 2.8  (0.7–6.7) | 0.3 ± 0.4  (0.1–1.4) | 8.3 ± 9.6  (0.9–26.0) | 7.3 ± 10.0  (1.0–26.1) | 0.3 ± 0.3 (0.1–1.4) | 7.2 ± 8.4 (0.3–26.0) | 6.3 ± 8.6 (0.7–26.1) |
| Aldosterone, pmol/L | 127.0 ± 177.0  (0–388) | 16.5 ± 33.0  (0–66) | 62.5 ± 125.0  (0–250) | 165.5 ± 255.1  (0–927) | 52.2 ± 76.8  (0–284) | 34.8 ± 47.9  (0–133) | 156.9 ± 235.7  (0–927) | 43.3 ± 69.3 (0–284) | 41.7 ± 70.4 (0–250) |
| Renin, mU/L | 74.0 ± 102.3  (6–224) | 54.5 ± 93.2  (4–194) | 57.1 ± 98.2  (0–204) | 23.7 ± 18.1  (3–68) | 59.6 ± 149.8  (0–533) | 66.6 ± 148.7  (3–533) | 34.9 ± 50.6 (3–224) | 58.3 ± 134.9 (0–533) | 64.2 ± 134.8 (0–533) |

All data are mean ± SD (range). Normal ranges are as follows: ACTH, 1.8–9.2 pmol/L; 11-deoxycortisol, 0–3.92 nmol/L, 11-deoxycorticosterone,
0.12–0.35 nmol/L (males) and 0.05–0.39 nmol/L (females); renin, not available; aldosterone, 55–250 pmol/L. SD, standard deviation

**Supplementary Figure 1. Absolute change in ACTH from baseline in the 17 patients who completed 22 weeks (safety analysis set)**

Note: Individual patient data are shown in the same order as in Figure 1 in the manuscript. Normal range: 1.1–11.1 pmol/L

The observed increase in mean ACTH levels was primarily driven by two patients. In one patient, who originally presented with an aggressive tumor with invasion of the left cavernous sinus at diagnosis that was treated with surgery and radiation, ACTH levels reached 581 pmol/L at week 22 and a peak of 1084 pmol/L at week 26 at an osilodrostat dose of 60 mg/day. Tumor size was not evaluable at baseline and week 22. In a second patient, the initial osilodrostat dose of 10 mg/day was stable from day 1 to week 22, and UFC values were <ULN at all occasions from weeks 2 to 22. ACTH levels increased from 6 pmol/L at baseline to 243 pmol/L at week 18, 196 pmol/L at week 22 and 231 pmol/L at week 26. The greatest tumor diameter increased from 3.3 to 5.0 mm (change 1.7 mm) from baseline to week 22. Tumor volume increased from 13.7 mm^3^ at baseline to 17.5 mm^3^ at week 22, representing a 28% increase. The patient did not experience any symptoms of compression of the optic chiasm from the tumor, or any symptoms to suggest adrenal insufficiency, except for fatigue.

**Supplementary Figure 2. Individual changes in testosterone levels during treatment (safety analysis set)**

Note: Each line represents an individual patient. Normal ranges are as follows: males, 8.7–38.2 nmol/L; females, 0.1–1.6 nmol/L

Estradiol, LH, and serum FSH levels in women were, in most cases, within the normal range; however, since data on the stage of the menstrual cycle and menopausal status were not collected, interpretation of these results is limited. In the five male patients, serum estradiol levels showed mild and transient increases above the normal range. In men, serum LH and FSH levels slightly decreased from baseline to week 22 (Supplementary Table 2). In females, there was a four-fold increase in LH and a 2.4-fold increase in FSH from baseline to week 22.

**Supplementary Table 2. LH and FSH values at baseline and week 22, by gender**

|  |  | **All patients** | |  |
| --- | --- | --- | --- | --- |
|  |  | **Baseline** | **Week 22** | **Change** |
| LH, U/L | Male | 3.2 ± 1.9 | 2.4 ± 1.8 | –0.8 ± 2.9 |
|  | Female | 2.4 ± 2.1 | 9.9 ± 17.0 | 7.8 ± 15.7 |
| FSH, U/L | Male | 5.9 ± 2.8 | 3.9 ± 2.9 | –2.0 ± 2.2 |
|  | Female | 7.6 ± 11.9 | 18.9 ± 37.1 | 11.3 ± 24.1 |

All data are mean ± SD. Normal ranges are as follows: LH males, 1.5–34.6 U/L; LH females,
0.5–76.3 U/L, depending on the stage of the menstrual cycle and menopausal status; FSH males, 1.6–8.0 U/L; FSH females, 1.5–116.3 U/L, depending on the stage of the menstrual cycle and menopausal status. Note: Information on the menstrual cycle and menopausal status was not collected

**Changes in pituitary tumor size**

**Supplementary Table 3. Pituitary tumor size (longest diameter) in evaluable^a^ patients at baseline and week 22 (safety analysis set)**

| **Age (years)/sex** | **Baseline diameter,^b^ mm** | **Week 22 diameter,^b^ mm** | **Change in diameter, mm** |
| --- | --- | --- | --- |
| 28/female | 3.3 | 5.0 | +1.7 |
| 51/female | 6.9 | 6.9 | 0 |
| 43/female | 5.0 | 4.0 | –1.0 |
| 35/female | 10.0 | 9.0 | –1.0 |
| 39/female | 7.0 | 6.0 | –1.0 |
| 29/male | 3.5 | 5.0 | +1.5 |

^a^Measurable tumor diameter at both baseline and week 22. ^b^Defined as the longest measurable diameter

**Safety and tolerability of osilodrostat**

Three serious AEs were reported in two patients. One patient had two serious AEs reported concurrently at week 34: gastroenteritis and QT prolongation. The QT prolongation was noted when the patient was hospitalized for gastroenteritis with dehydration; it did not reappear when treatment was resumed. Another patient was hospitalized during week 14 for polyuria and polydipsia, most likely related to diabetes insipidus, with uncontrolled Cushing’s disease (UFC levels increased from within the normal range [57 nmol/24h] at week 8 to 389 nmol/24h at the time of hospitalization and 527 nmol/24h at week 22). Osilodrostat dose changes in this patient are already described on page 3 of the Supplementary Appendix.

**Supplementary Table 4. Details on patients with adrenal insufficiency reported as an AE**

| **Pt** | **Day AE reported** | **Duration of AE, days** | **UFC when AE first reported, nmol/24h** | **Morning serum cortisol when AE first reported, nmol/L** | **Other symptoms reported** | **Osilodrostat dose changes** | **GC replacement therapy** | **Notes** |
| --- | --- | --- | --- | --- | --- | --- | --- | --- |
| 1 | 18 and 51 | 3 and 9, respectively | 116 and 31, respectively (WNR) | 196 and 232, respectively (WNR) | Fatigue | Interrupted for 1–2 days on three occasions. Decreased from 40 to  10 mg/day | None | Vital signs normal and no hypotension. Potassium was  3.3 mmol/L (normal range:  3.4–4.8 nmol/L) on day 56 and  was normalized at day 70. Sodium, calcium and magnesium levels were normal on days 14 and 56 |
| 2 | 180 | 11 | 10 (<LLN) | 119 (<LLN) | Dizziness, asthenia, depression | Decreased from 20 to  10 mg/day | None | Vital signs and electrolytes normal. No hypotension. Hypomagnesemia (0.70 mmol/L; normal range 1.8–2.5) was reported as an AE (day 182) |
| 3 | 57 | 70 | 5 (<LLN) | 61 (<LLN) | Nausea, tachycardia (days 58  and 69, respectively) | Decreased from 10 to  2 mg/day | None | Vital signs, electrolytes, magnesium, and calcium normal. No hypotension |
| 4 | 182 | 60 | 11.7 (WNR) | 72 (<LLN) | None | Decreased from 60 to  10 mg/day; later interrupted for a few weeks | None | From baseline to day 182: SBP decreased from ~138 to ~100 mmHg; heart rate increased from ~90 to  122 bpm. No hypotension. Electrolytes, magnesium, and calcium normal |
| 5 | 205 | Unknown | 6.4 (<LLN) | 160 (WNR) | Syncope, malaise | Decreased from 10 to  2 mg/day | None | SBP and DBP decreased from ~120/83 on day 1 to ~110/60 mmHg on day 210. Electrolytes, magnesium, and calcium normal |
| 6 | 37 | Unknown | 3.5 (<LLN) | 135 (WNR) | Not reported | Decreased from 7 to  4 mg/day | Dex initiated on day 56 | Vital signs, electrolytes, magnesium, and calcium normal. No hypotension |

Normal ranges are as follows: UFC, 11–138 nmol/24h; morning serum cortisol, 127–567 nmol/L. DBP, diastolic blood pressure; Dex, dexamethasone;
GC, glucocorticoid; Pt, patient; SBP, systolic blood pressure; WNR, within normal range

**References**

1. American Heart Association. Understanding blood pressure readings. 2014. Available at: <http://www.heart.org/HEARTORG/Conditions/HighBloodPressure/AboutHighBloodPressure/Understanding-Blood-Pressure-Readings_UCM_301764_Article.jsp>.
